# Proton NMR-Based Metabolite Analyses of Archived Serial Paired Serum and Urine Samples from Myeloma Patients at Different Stages of Disease Activity Identifies Acetylcarnitine as a Novel Marker of Active Disease

**DOI:** 10.1371/journal.pone.0056422

**Published:** 2013-02-19

**Authors:** Alessia Lodi, Stefano Tiziani, Farhat L. Khanim, Ulrich L. Günther, Mark R. Viant, Gareth J. Morgan, Christopher M. Bunce, Mark T. Drayson

**Affiliations:** 1 School of Cancer Sciences, The University of Birmingham, Birmingham, United Kingdom; 2 Department of Nutritional Sciences, The University of Texas at Austin, Austin, Texas, United States of America; 3 Dell Pediatric Research Institute, The University of Texas at Austin, Austin, Texas, United States of America; 4 School of Biosciences, The University of Birmingham, Birmingham, United Kingdom; 5 Institute of Cancer Research, Royal Marsden NHS Foundation Trust, London, United Kingdom; 6 School of Immunity and Infection, The University of Birmingham, Birmingham, United Kingdom; George Washington University, United States of America

## Abstract

**Background:**

Biomarker identification is becoming increasingly important for the development of personalized or stratified therapies. Metabolomics yields biomarkers indicative of phenotype that can be used to characterize transitions between health and disease, disease progression and therapeutic responses. The desire to reproducibly detect ever greater numbers of metabolites at ever diminishing levels has naturally nurtured advances in best practice for sample procurement, storage and analysis. Reciprocally, since many of the available extensive clinical archives were established prior to the metabolomics era and were not processed in such an ‘ideal’ fashion, considerable scepticism has arisen as to their value for metabolomic analysis. Here we have challenged that paradigm.

**Methods:**

We performed proton nuclear magnetic resonance spectroscopy-based metabolomics on blood serum and urine samples from 32 patients representative of a total cohort of 1970 multiple myeloma patients entered into the United Kingdom Medical Research Council Myeloma IX trial.

**Findings:**

Using serial paired blood and urine samples we detected metabolite profiles that associated with diagnosis, post-treatment remission and disease progression. These studies identified carnitine and acetylcarnitine as novel potential biomarkers of active disease both at diagnosis and relapse and as a mediator of disease associated pathologies.

**Conclusions:**

These findings show that samples conventionally processed and archived can provide useful metabolomic information that has important implications for understanding the biology of myeloma, discovering new therapies and identifying biomarkers potentially useful in deciding the choice and application of therapy.

## Introduction

Multiple myeloma (MM) is a malignancy of differentiated B cells (plasma cells) which form and accumulate in the bone marrow environment [1], [2]. These cells manufacture and secrete large quantities of monoclonal whole immunoglobulin (Ig) and free Ig light chain (flc) into blood [3]. The flc are filtered into urine and often cause renal impairment (RI) at some point during disease with 30% of patients presenting with RI at diagnosis [4]. MM also dysregulates bone turnover causing lytic bone lesions and fractures, and impairs haemopoiesis usually resulting in anaemia [5], [6]. MM induces suppression of normal antibody production which with other less clear mechanisms leads to severe reduction in immunocompetence and serious infection.

The levels of monoclonal Ig and flc in the blood and urine increase as disease progresses and decrease as disease responds to treatment. Survival for IgG patients is better than patients with IgA monoclonal Ig. The presence and level of flc greatly increases the risk of RI and RI is associated with worse survival during periods of active disease. RI results in elevated beta2 microglobulin levels as well as elevated serum creatinine levels. Beta2 microglobulin also reflects disease load and is the strongest prognostic blood biomarker for overall survival for myeloma patients. However, to date, prognostic markers in myeloma do not reliably predict outcome for individual patients, rather they are used to ensure that randomisation into treatment arms of large trials is balanced for patient prognosis [7].

In 2008 a total of 4,400 new cases and 2,660 deaths from MM were reported in the UK. From the 1960s treatment with melphalan and prednisolone achieved a median survival of 2–2.5 years. By the 1990s widespread use of high dose melphalan and autologus stem cell rescue in younger fitter patients improved median survival to 3–4 years [8]. New therapies incorporating combinations of corticosteroids and conventional chemotherapy agents with immunomodulatory drugs (IMiDs; thalidomide and lenalidomide), and proteasome inhibitors (PIs, bortezomib) have increased median survival to 4–5 years [9]. Current treatment strategies utilise induction regimens combining a mixture of conventional drugs plus IMiDs or proteasome inhibitors and in younger fitter patients high dose melphalan [10], [11]. The aim is to achieve stable remission with a maximal reduction in tumour load and delayed relapse from remission.

Recent evidence indicates over 90% of newly diagnosed myeloma clones are derived from a premalignant clonal expansion of plasma cells called Monoclonal Gammopathy of Undetermined Significance (MGUS) [12], [13]. Progression from MGUS to active myeloma is characterized by proliferation of the malignant plasma cells but is poorly understood. The genetic translocations associated with myeloma are commonly found in MGUS [14] and there is a need to identify biomarkers that indicate the mechanisms and risk of disease progression. Myeloma itself remains very heterogeneous and unpredictable in individual patient outcome with an urgent need for biomarkers by which treatment can be stratified. A substantial proportion of patients do not reach a stable remission and die early from their disease. In less than half of patients reaching remission is a complete tumour response achieved and yet many partial responders enjoy stable asymptomatic remissions without further therapy for periods as long as those patients who achieve complete remissions. These partial responders appear to have returned to a state similar to MGUS.

New biomarkers are urgently required to identify risk of progression from MGUS to myeloma, to stratify patients for induction therapies, identify risk of progression from remission, and to stratify patients for maintenance and subsequent therapies. Proton nuclear magnetic resonance (NMR) spectroscopy-based metabolomics can be used to determine complex small molecule mixtures in both blood serum and urine and has emerged as a valuable approach to dissecting disease processes [15]–[23]. Alongside the development of advanced NMR technologies to measure the complex metabolite mixtures in patient material and the development of software to analyze the derived data, there have also been major advances in ‘best practice’ protocols for the collection, storage and preparation of samples for analysis [24], [25].

The improved reproducibility achieved by these protocols has naturally cast doubt upon the value of extant tissue and biofluid archives collected prior to their development. However, existing archives and especially those associated with large randomised clinical trials, are allied to large and detailed amounts of patient data. In addition, further data from genomic and/or cytogenetic analyses are commonly available. Given typical patient accrual rates and the need to wait seven or more years to assess overall survival rates it will take ten years to replace many currently available archives with equally rich replacements in which sample procurement, storage and processing has been optimised. Furthermore, pragmatic issues of time and fiscal restraints render it unlikely that current ‘best practice’ practice for sample procurement and storage will ever be achieved in the settings of large scale phase III trials or routine clinical practice. It is important therefore that the community adopts a more empirical and scientific approach to determining the value of archived material for the timely discovery of new biomarkers for better disease management and understanding of disease biology.

To address these issues, we have applied proton NMR-based metabolomics to investigate the metabolic profiles of archived blood serum and urine from 32 patients as they entered into the United Kingdom Medical Research Council Myeloma IX trial with newly diagnosed myeloma requiring therapy, when they had achieved asymptomatic remission, and when some had subsequently relapsed and some remained asymptomatic. These 32 patients represent a small subset of 1,970 Myeloma IX patients for whom there are multiple longitudinal samples associated with parallel clinical data and also time of entry gene expression array data.

Despite the challenging history and small number of samples analyzed we were able to detect metabolic signatures predictive of disease state. In particular we have identified a previously unknown association of elevated carnitine and acetylcarnitine as a signature of active myeloma. Our findings have important implications both for the future study of myeloma but also for the broader application of metabolomics to other patient material archives in other diseases.

## Materials and Methods

### Blood Serum and Urine Samples

Archived samples from 32 myeloma patients that participated in the United Kingdom Medical Research Council Myeloma IX trial were analyzed. This trial was a multicenter, phase 3, trial registered at www.isrctn.org as ISRCTN68454111, recruiting patients from 2003 to 2007 from 120 centres. The trial was approved by the North West Multi-centre Research Ethics Committee, and by local review committees at all participating centres. All patients provided written informed consent for additional blood and urine samples to be sent for central laboratory analysis and storage for future research on myeloma. Permission for this specific research was granted by the Life and Health Sciences Ethical Review Committee of the University of Birmingham, UK. The samples were collected at different times in the day according to outpatient attendance time and not fasted. Urine was collected into 25 ml universal containers with sodium azide and blood into standard red topped glass vacutainers for clotted blood that did not contain gel. They were sent via the Royal Mail postal service and thus spent 1–3 days at ambient temperature before arriving at the Trial Immunodiagnostic Central Laboratory. On arrival samples were centrifuged, serum and urine aliquoted and refrigerated at 4°C for 2–3 weeks before storage frozen at −20°C in screw topped, o-ringed, polypropylene tubes.

### Patient Characteristics

Patients’ characteristics are included in Table 1. Patients were selected to be the first 32 patients for whom Affymetrix gene array data on purified myeloma cells at diagnosis were available and for whom there were paired serum and urine samples available at diagnosis before treatment, in first remission, and a subsequent sample either when still in remission or at first relapse. Eighteen patients received intensive therapy [26] and 14 patients received non intensive therapy [27]. Patients were assigned to one of these two treatment groups according to their general health and fitness to tolerate intensive chemotherapy. The course of myeloma disease is very heterogenous between patients, although overall survival is better in fitter patients receiving intensive therapy. Time from diagnosis to remission was between 5–12.5 (median 8) months for intensive patients and between 4–13 (median 8) months for non intensive patients. Time from remission to relapse, for those who did relapse, was between 3–34 (median 26) months for intensive patients and between 3–34 (median 5.5) months for non intensive patients. We would expect to need to analyse samples from at least 600 patients to properly assess the prognostic value of our metabolomic findings for the outcomes overall survival and progression free survival. However, this aspect was disregarded in the classification of the patients for statistical analysis as we aimed at determining general metabolic signatures of active disease versus remission. Renal function usually improves as patients enter into remission and may deteriorate at relapse with associated changes in beta2 microglobulin and creatinine levels. However, changes in renal function do not occur in a third of patients and changes in renal function also occur independently of changes in disease activity. For patients in group A, mean (± standard deviation) serum creatinine and beta2 microglobulin levels were 104±46 µmol/l and 4.9±3.4 mg/l, respectively. For group B, mean serum creatinine and beta2 microglobulin levels were 96±33 µmol/l and 2.9±1.0 mg/l. For group C1, mean serum creatinine and beta2 microglobulin levels were 87±21 µmol/l and 2.3±1.0 mg/l. For group C2, mean serum creatinine and beta2 microglobulin levels were 114±32 µmol/l and 3.9±2.2 mg/l.

**Table 1:** Patients characteristics.

Treatment	Response in C	Age (yrs)	Sex	Paraprotein type	Serum beta2 microglobulin (mg/l)			Serum creatinine (µmol/l)		
					A	B	C	A	B	C
Intensive	Remission	62	M	GLO	3.1	2.4	2.1	117	107	92
Intensive	Remission	66	M	GKU	3.8	2.1	2.2	92	95	102
Intensive	Remission	52	F	GK0	3.5	2.1	1.8	78	75	75
Intensive	Remission	54	F	NS	1.0	2.4	2.3	43	63	84
Intensive	Remission	58	F	KU0	1.3	1.9	1.1	45	51	63
Intensive	Remission	52	M	AK0	2.7	1.9	1.6	106	84	103
Intensive	Remission	51	F	DLU	7.9	1.7	1.5	112	54	47
Intensive	Remission	57	F	ALU	2.7	1.8	1.9	84	76	85
Intensive	Remission	54	M	GLU	17.9	2.8	1.9	237	77	74
Intensive	Relapse	57	F	KUS	7.9	4.4	2.4	115	59	106
Intensive	Relapse	53	M	GLU	8.8	2.6	3.5	122	114	124
Intensive	Relapse	58	M	GKU	3.7	3.0	2.6	95	75	111
Intensive	Relapse	53	M	GK0	3.3	2.8	2.3	79	83	119
Intensive	Relapse	58	M	GKU	3.1	2.4	2.1	86	87	88
Intensive	Relapse	46	M	AKU	1.4	2.9	4.1	65	109	120
Intensive	Relapse	61	M	AKU	3.2	3.3	2.9	98	102	111
Intensive	Relapse	52	M	AKU	1.8	2.4	2.1	62	82	81
Intensive	Relapse	52	F	ALO	3.3	3.0	1.8	75	113	77
Non Int.	Remission	80	F	GLO	3.8	4.0	4.0	57	69	75
Non Int.	Remission	71	M	KUS	3.7	3.7	4.7	103	164	120
Non Int.	Remission	74	M	ALU	7.2	2.3	2.1	116	77	88
Non Int.	Remission	76	M	GKU	7.3	3.0	2.9	133	94	119
Non Int.	Relapse	69	M	DLU	5.2	5.0	7.2	154	153	187
Non Int.	Relapse	74	M	GKU	6.7	3.0	4.3	135	133	137
Non Int.	Relapse	67	M	GLU	3.6	2.0	2.4	122	114	97
Non Int.	Relapse	73	F	KUS	4.4	3.1	3.8	118	61	89
Non Int.	Relapse	78	F	ALO	5.9	4.6	4.4	83	86	76
Non Int.	Relapse	74	F	AKU	11.2	3.3	10.7	165	102	141
Non Int.	Relapse	75	F	GLU	3.6	5.5	4.3	68	184	156
Non Int.	Relapse	71	M	GKU	3.1	3.8	3.2	78	75	85
Non Int.	Relapse	70	F	GKO	4.8	2.0	5.6	57	61	82
Non Int.	Relapse	68	M	ALU	5.7	2.8	3.7	232	145	154

Footnotes table 1. Serum reference ranges:- beta2 microglobulin 0.5–4.0 mg/l; serum creatinine 45–110 µmol/l (0.5 to 1.2 mg/dl). Paraprotein types detected by immunofixation of serum and urine: GLO IgG lambda, no flc in urine; GKU IgG kappa, flc in urine; NS non secretory, no Ig detected in blood or urine; KUO kappa flc only detected in urine but not serum; AKU IgA kappa, kappa flc in urine; ALO IgA lambda, no flc in urine; KUS kappa flc only detected in serum and urine; ALU IgA lambda, flc in urine; DLU IgD lambda, flc in urine.

### NMR Sample Preparation

Frozen blood serum and urine samples were allowed to defrost completely on ice. The preparation of blood serum samples was performed as previously described [24]. Briefly, approximately 0.5 ml of human serum was filtered (Nanosep 3K OMEGA, Pall Corporation, MI) at 4°C at 10,000 rpm to remove the protein and lipid fractions. 420 µl of filtered serum (volume adjusted with water, when needed) were mixed with 120 µl of phosphate buffer 0.5 M (pH 7.0±0.1) containing 0.75% w/v sodium azide and 60 µl of 5 mM TMSP in D_2_O (99.9% pure; GOSS Scientific Instruments Ltd, Essex UK) and transferred to an NMR tube. Urine samples were prepared as previously described [28]. Briefly, 540 µl of urine (at room temperature) was mixed with 180 µl of 0.4 M phosphate buffer (pH 7; containing 0.75% w/v sodium azide). Samples were allowed to stand for 20 minutes and centrifuged at 13,000 rpm for 3 min. The supernatant was transferred to a clean tube and the pH measured and adjusted to 7. Samples were allowed to stand for 20 minutes and centrifuged again. These steps were repeated until precipitation was no longer observed following centrifugation and the supernatant pH = 7–7.1. 540 µl of the resulting sample were mixed with 60 µl of 5 mM TMSP in D_2_O.

### NMR Data Acquisition and Processing

A 500 MHz Bruker (Bruker Biospin, Rheinstetten, Germany) spectrometer equipped with a cryogenically cooled probe was used for one dimensional (1D) proton (^1^H) NMR data acquisition of both serum and urine samples. 1D spectra were acquired using excitation sculpting (“zgesgp”) for suppression of water resonance [29]. 1D spectra were acquired with a long relaxation delay of 15 s and a 30° flip angle to guarantee near complete longitudinal relaxation, and with a spectral width of 5 kHz and 256 transients. Exponential multiplication (lb = 0.5) and zero filling (to double the number of points and improve the subsequent spectral alignment) were performed prior to Fourier transformation. Spectra were phased and aligned. Signals arising from water and TMSP were excluded. Spectra were normalized according to the probabilistic quotient normalization method [30] and segmented into ‘bins’ of width 0.0015 ppm. A generalized-log transformation was applied prior to conducting the multivariate statistical analysis [28]. The NMR datasets were processed using NMRLab [31] in the MATLAB programming environment (The MathWorks, Inc., Natick, MA). NMR resonances of metabolites were assigned using the Chenomx NMR Suite (version 5.0; Chenomx Inc., Edmonton, Canada). Selected signals were quantified (for 1D fully relaxed spectra) using peak deconvolution (Topspin 2.1, Bruker Biospin). The concentrations of metabolites evaluated against the TMSP peak (added to the samples at 0.5 mM final concentration). The dilution steps entailed by NMR sample preparation (in particular, addition of water, phosphate buffer and deuterium oxide) were taken into account and the metabolite concentrations included herein are representative of the concentration in the original serum samples. Concentrations are reported as mean values ± SEM and the reported statistical significance is based on the Kruskal-Wallis one-way ANOVA (MATLAB). Receiver operating characteristic (ROC) curve was calculated using MATLAB.

### Multivariate Statistical Analysis

Unsupervised (principal components analysis; PCA) and supervised (partial least squares discriminant analysis; PLS-DA) multivariate analyses were performed using PLS-Toolbox (Version 4.1; Eigenvector Research, Manson, WA). All the models were built using the indicated number of classes (according to the classification outlined below) and the optimal number of latent variables (LV) determined by the minimum classification error. Data pre-processing was always performed using mean centering and orthogonal signal correction (OSC [32]). All the models built using OSC-PLS-DA were validated using permutation tests to assess the statistical significance of the model’s predictive power. Briefly, 1000 internally cross validated (using Venetian blind, with number of splits equal to the square root of the sample size), “permuted” models were built using random permutation of the sample labels [33]. The classification errors of the 1000 “permuted” models were compared to the average of 1000 calculations of the model for the real (without scrambling of the sample labels) dataset. The predictivity of the model (as an average across all the classes) was considered significant when less than 50 out of the 1000 “permuted” models had classification error values lower that the average one obtained for the “real” dataset (5% significance level).

### Patient Classification for Multivariate Statistical Analysis of NMR Data

For all the multivariate statistical analyses patients were classified as follows. All the samples obtained at diagnosis were included in one group (A). All the samples obtained after the end of chemotherapy from patients in remission were included in group B (samples obtained at least 1 month after the end of chemotherapy for the non-intensive group and at least 3 months after the end of chemotherapy for the intensive group). Finally, samples collected several months (at least 3) after the end of chemotherapy were included in group C. Samples obtained at this time point were both from patients that were still in remission and from patients that had relapsed. Therefore group C was further stratified into group C1, including patients in remission, and group C2, including patients with relapsed disease.

## Results

### Preliminary Screening of NMR Spectra of Blood Serum and Urine Samples

**Figure 1:**
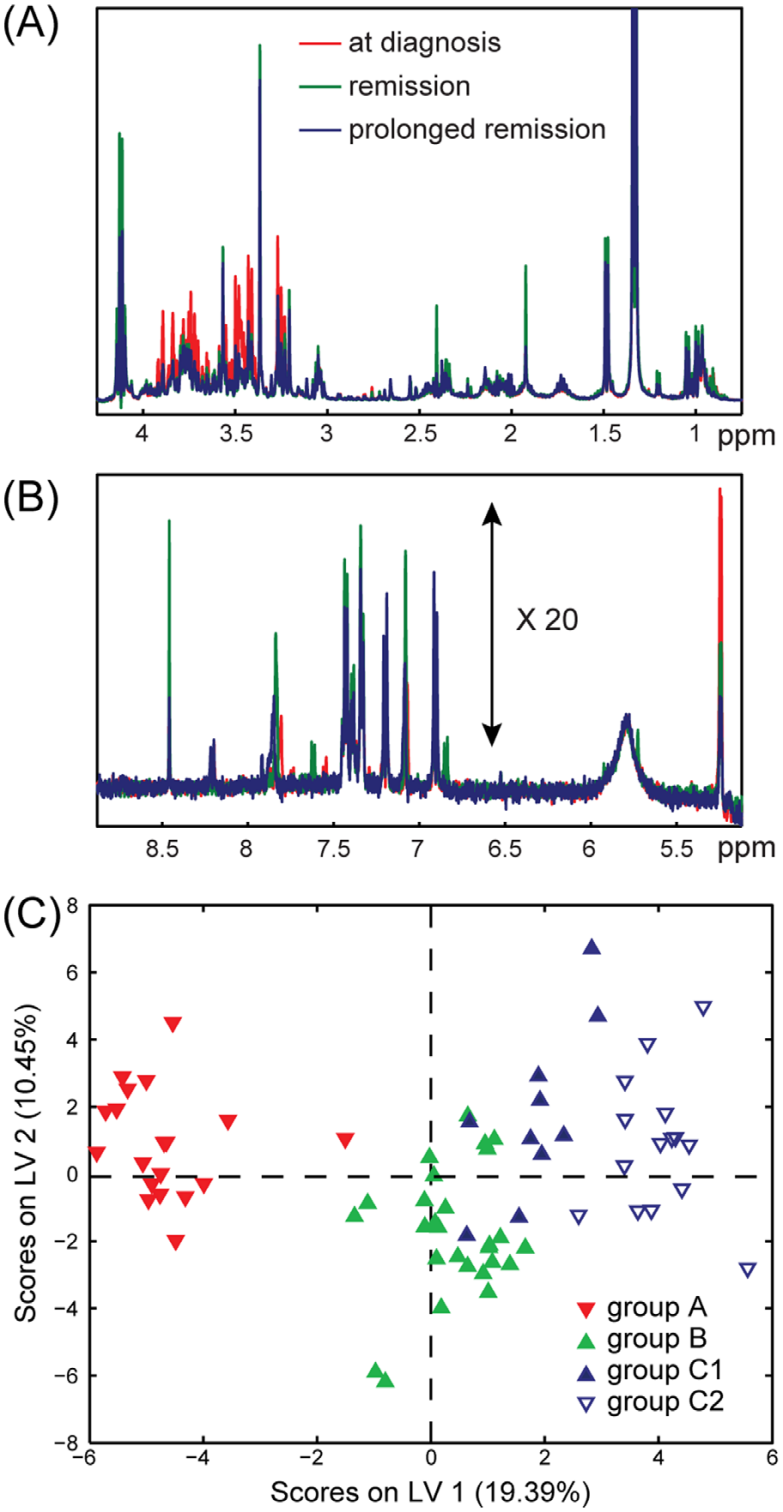
Proton NMR spectra and Partial Least Squares Discriminant Analysis of blood serum samples. Representative sections of proton NMR spectra at the diagnosis (red), remission (green) and prolonged remission (blue) of one multiple myeloma patient. (**A**) up-field region (0.75–4.25 ppm), (**B**) down-field region (5.1–8.9 ppm), 20 times increased intensity compared to (A). (**C**) Scores plot obtained from OSC-PLS-DA performed on the NMR spectra of 71 blood serum samples. Group A (solid red, 19 samples): patients at diagnosis; group B (solid green, 27 samples): patients after chemotherapy; group C1 (solid blue, 10 samples): sustained remission and group C2 (empty blue, 15 samples): in relapse after chemotherapy.

One-dimensional proton NMR spectra were acquired on a total of 71 blood serum and 73 urine samples from 32 myeloma patients. Representative sections of NMR spectra for one patient are shown in **Figures 1A–B**. A preliminary analysis of the acquired NMR spectra revealed that 6 urine samples (1 from group A, 1 from B, and 4 from C1–C2) were outliers, the cause of which was determined to be considerable amounts of glucose. These were subsequently excluded from the multivariate analyses. Therefore, all the models were built using 71 blood serum samples (19, 27, 10 and 15 samples for groups A, B, C1 and C2, respectively) and 67 urine samples (21, 24, 10 and 12 samples for groups A, B, C1 and C2, respectively). PCA was performed on both the blood serum and urine samples and indicated a partial classification of samples in the different groups but no clear separation (**Figure S1**). The initial supervised multivariate statistical analysis (PLS-DA) indicated that the NMR resonances arising from the drug acetaminophen and its metabolic products (acetaminophen glucuronide (AG), acetaminophen sulphate (AS) and N-acetyl-L-cysteine acetaminophen (NAC)) were found in variable amounts in both urine (considerable amounts leading to rather high intensity signals) and blood serum (very low NMR signal intensities) of several patients [34]. As we sought to analyse only the endogenous metabolome of the disease process these peaks were considered as confounding factors in the classification and were therefore excluded from the analyses.

### Multivariate Statistical Analysis of NMR Spectra of Blood Serum Samples

**Figure 1C** shows the scores plot obtained from the OSC-PLS-DA of the 71 blood serum samples. The OSC-PLS-DA model was built using 4 classes (A, B, C1 and C2) and 2 LVs. The validation using permutation testing identified that the predictivity of the model was significant (p∼0.027), thereby justifying the need for a more detailed analysis of the data. Sensitivity and specificity values calculated for cross-validated OSC-PLS-DA using Receiver Operating Characteristic (ROC) curves were as follows: 47.4% and 90.4% for group A, 74.1% and 65.9% for group B, 60.0% and 77.7% for group C1, and 46.7% and 76.9% for group C2, respectively. To better investigate the metabolites with the strongest discriminating power between the different disease time-points, PLS analyses were repeated for blood serum samples considering only 2 groups per analysis as indicated.

### OSC-PLS-DA Comparing Patient Blood Serum at Diagnosis Versus Post-treatment Remission

**Figure 2:**
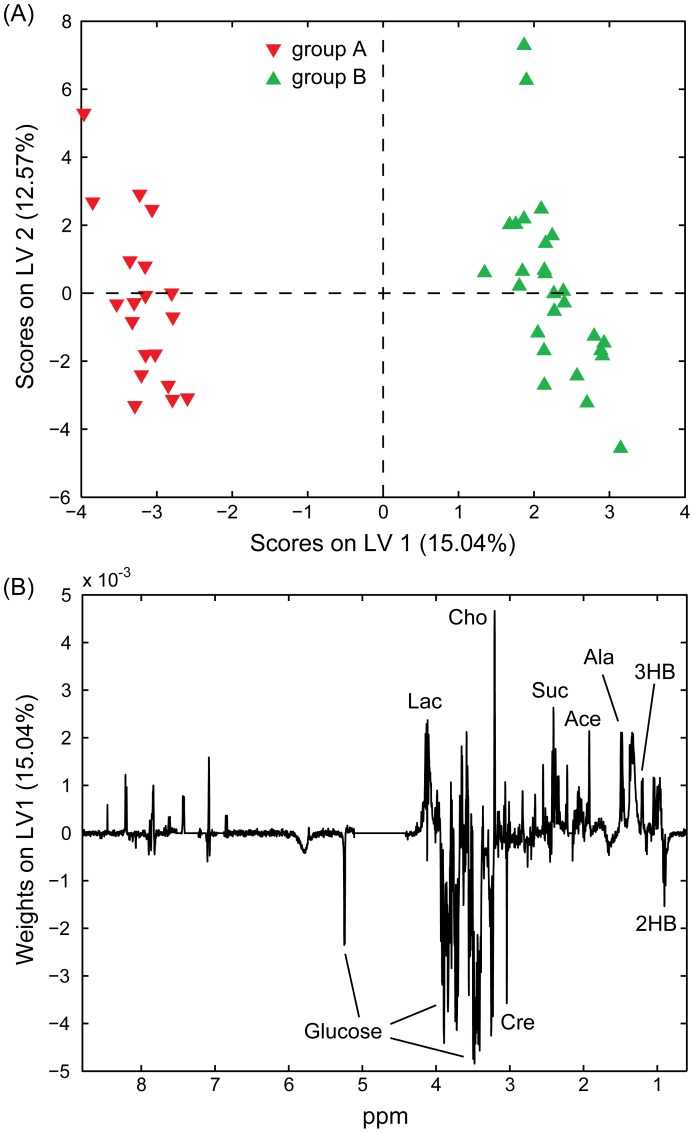
Partial Least Squares Discriminant Analysis of NMR spectra acquired on blood serum samples. Scores (**A**) and weights (on LV1; **B**) plots obtained from OSC-PLS-DA performed on the NMR spectra of 46 blood serum samples. Group A (solid red, 19 samples): patients at diagnosis; Group B (solid green, 27 samples): patients after chemotherapy. Lac: lactate; Cho: choline; Cre: creatinine; Suc: succinate; Ace: acetate; Ala: alanine; 3HB: 3-hydroxybutyrate; 2HB: 2-hydroxybutyrate.

We first compared blood serum samples from patients at diagnosis (group A) and when in remission (group B). The scores and weights plots from this 2-LV (optimised) model are included in **Figure 2** (**A** and **B**, respectively). Sensitivity and specificity values on cross-validated analysis were 63.2% and 66.7% for group A, and 66.7% and 63.1% for group B. The predictivity of the model was highly significant (p∼0.004) and the analysis of the weights indicated that the main blood serum metabolic differences between patients at diagnosis and in post treatment remission are higher levels of glucose, creatinine and 2-hydroxybutyrate in patients at diagnosis (group A) and elevated concentrations of succinate, 2-hydroxyisobutyrate, 3-hydroxybutyrate, alanine and choline in patients that are in remission (group B) after receiving treatment.

### OSC-PLS-DA for Comparing Patient Blood Serum at Diagnosis Versus Long After Treatment (Either in Remission or Relapsed)

**Figure 3:**
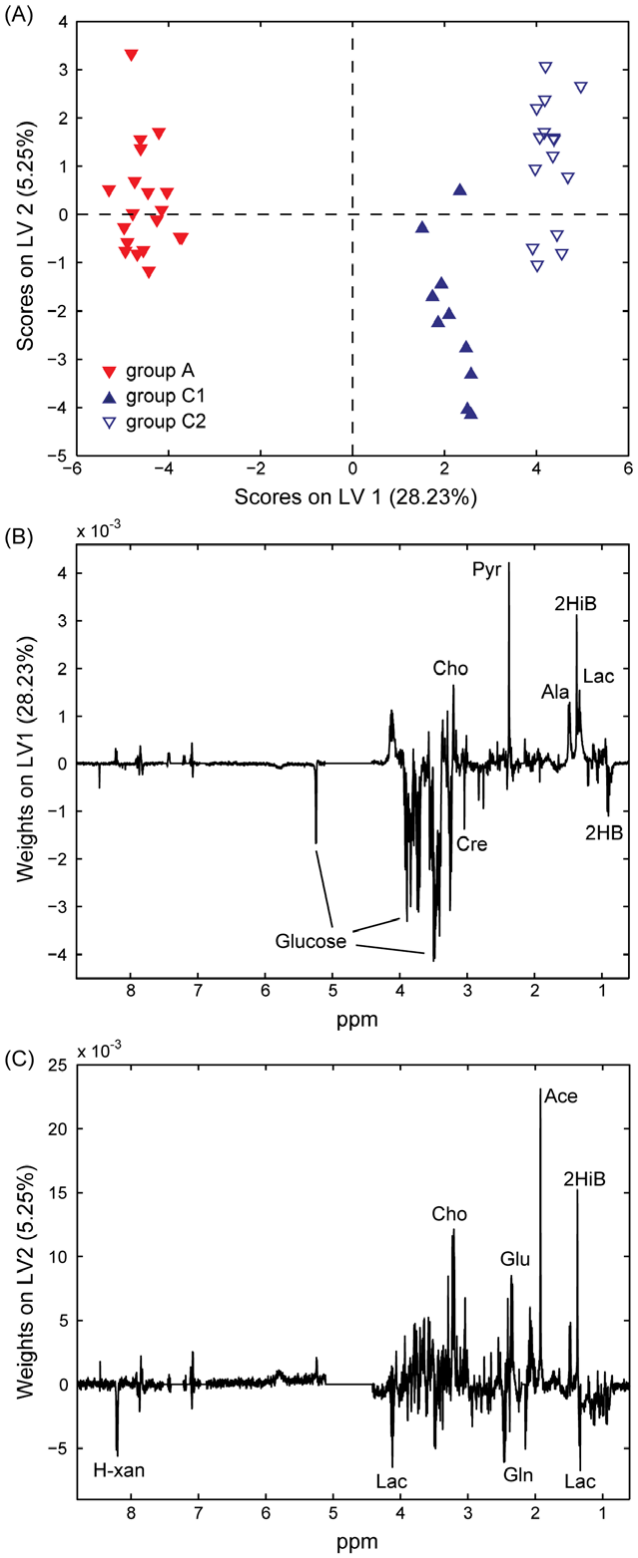
Partial Least Squares Discriminant Analysis of NMR spectra acquired on blood serum samples. Scores (**A**) and weights (on LV1, **B**, and LV2, **C**) plots obtained from OSC-PLS-DA performed on the NMR spectra of 44 blood serum samples. Group A (solid red, 19 samples): patients at diagnosis; group C1 (solid blue, 10 samples): sustained remission and group C2 (empty blue, 15 samples): in relapse after chemotherapy. Cho: choline; Cre: creatinine; Pyr: pyruvate; Ala: alanine; 2HiB: 2-hydroxyisobutyrate; Lac: lactate; 2HB: 2-hydroxybutyrate. H-xan: hypoxantine; Glu: glutamate; Gln: glutamine; Ace: acetate.

OSC-PLS-DA for the discrimination of groups A and C (3 classes, due to the distinction between classes C1, patients in remission, and C2, in relapse) was performed optimally using 3 LVs. Once again the model’s predictivity was demonstrated to be significant (p∼0.019) based upon permutation testing. Sensitivity and specificity values were 63.2% and 72.0% for group A, 30.0% and 79.4% for group C1, and 46.7% and 75.9% for group C2. The scores and weights plots are included in **Figure 3**. The LV1 weights (**Figure 3B**) indicate that glucose, creatinine and 2-hydroxybutyrate are present in higher concentration in the blood serum of patients at diagnosis (group A) while pyruvate, 2-hydroxyisobutyrate, choline, alanine, and lactate are decreased. Moreover, **Figure 3A** shows the layering of the subclasses along LV2, with C1 distributing mostly with negative LV2 scores and groups A and C2 (the active disease groups) towards positive LV2 scores. The LV2 weights (**Figure 3C**) indicated that lactate, glutamine and hypoxanthine were among the most important discriminant metabolites (higher in C1) while acetate, glutamate, 2-hydroxyisobutyrate and choline were more abundant in samples with positive LV2 scores.

The comparisons between patients at diagnosis (group A) and either C1 or C2 groups were also performed (**Figure S2**). Permutation testing of model predictivity yielded a significant result for the comparison of groups A-C1 (p∼0.027), and approached significance for A-C2 (p∼0.053). Sensitivity and specificity values for comparison of groups A versus C1 were 78.9% and 60.0% for group A, and 60.0% and 78.9% for group C1, while for comparison of groups A versus C2 were 78.9% and 66.7% for group A, and 80.0% and 73.7% for group C2.

This result might indicate an important underlying similarity between the blood serum metabolome of patients with active disease before and after treatment (in relapse). However, admittedly, the very limited number of samples from patients who relapsed after treatment (C2, 15 samples) might play a role in the inability to build a model with significant predictivity. Among the most important metabolites discriminating A and C1 (but not A and C2) were acetate, glutamate, succinate, creatinine and betaine (higher in A), and glutamine, lactate and hypoxanthine (higher in C1).

### OSC-PLS-DA for Comparing Patient Blood Serum during Initial Remission Versus After Sustained Remission or Relapsed

**Figure 4:**
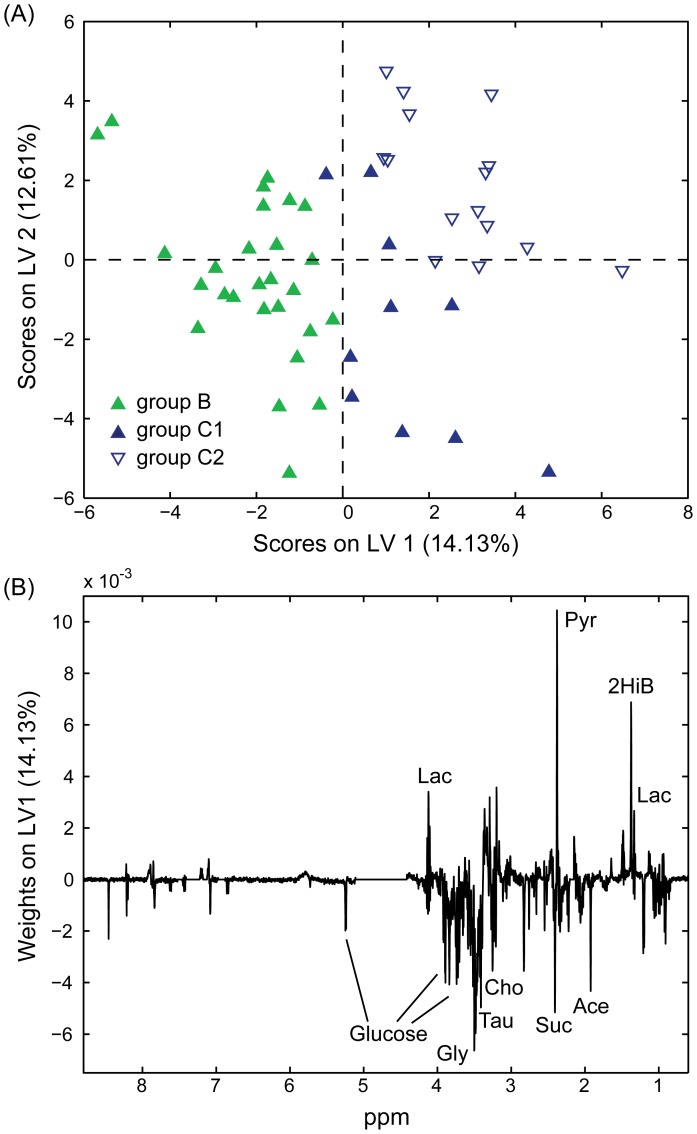
Partial Least Squares Discriminant Analysis of NMR spectra acquired on blood serum samples. Scores (**A**) and weights (on LV1; **B**) plots obtained from OSC-PLS-DA performed on the NMR spectra of 52 blood serum samples. Group B (solid green, 27 samples): patients after chemotherapy; group C1 (solid blue, 10 samples): sustained remission; group C2 (empty blue, 15 samples): in relapse after chemotherapy. Lac: lactate; Gly: glycine; Tau: taurine; Cho: choline; Suc: succinate; Pyr: pyruvate; Ace: acetate; 2HiB: 2-hydroxyisobutyrate.

OSC-PLS-DA for the discrimination of post-treatment remission samples (B) and those of patients in prolonged remission (C1) or relapse (C2) was optimised using 4 LVs. The predictivity of this model was significant (permutation testing, p∼0.038). The scores plot (**Figure 4A**), shows patients in remission (C1) clustering closer to group B. Sensitivity and specificity values of the model were 65.4% and 59.3 for group B, 27.3% and 76.2% for group C1, and 43.8% and 70.3% for group C2, respectively. The weights plot (**Figure 4B**) obtained from this analysis indicates that acetate, glycine, succinate and choline are present in higher concentration in the blood serum of patients in earlier post-treatment remission (B) while pyruvate and 2-hydroxyisobutyrate, were decreased.

**Figure 5:**
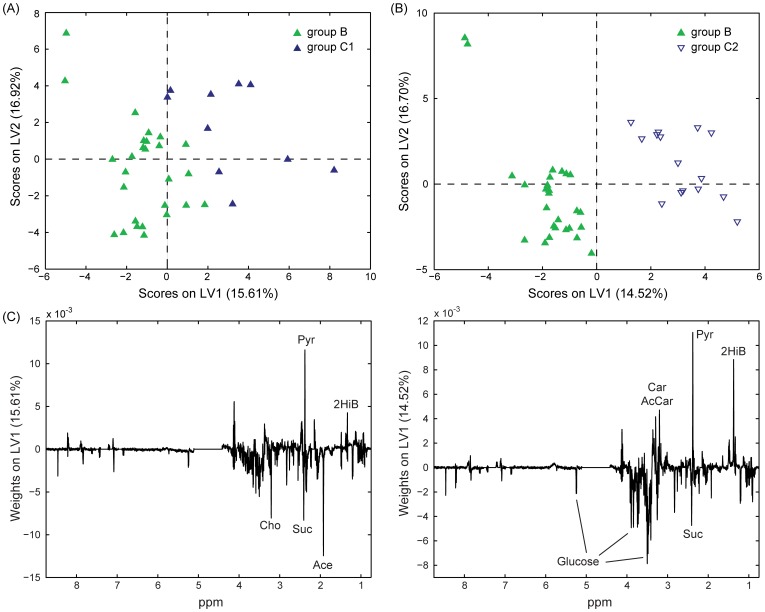
Partial Least Squares Discriminant Analysis of NMR spectra acquired on blood serum samples. Scores (**A** and **B**) and weights (on LV1; **C** and **D**) plots obtained from OSC-PLS-DA performed on the NMR spectra of 37 and 42 blood serum samples for the comparison of groups B versus C1 (**A** and **C**) and B versus C2 (**B** and **D**). Group B (solid green, 27 samples): patients after chemotherapy; group C1 (solid blue, 10 samples): sustained remission; group C2 (empty blue, 15 samples): in relapse after chemotherapy. Cho: choline; Suc: succinate; Pyr: pyruvate; Ace: acetate; 2HiB: 2-hydroxyisobutyrate; Car: carnitine; AcCar: acetylcarnitine.

In addition, we also compared group B samples separately with either C1 or C2 samples, and again permutation testing of these models indicated significant predictivity p∼0.031 and p∼0.023, respectively (scores plots in **Figures 5A and B** **)**. Sensitivity and specificity values for comparison of groups B versus C2 were 81.5% and 60.0% for group B, and 60.0% and 81.5% for group C1, while for groups B versus C2 were 70.4% and 46.7% for group B, and 46.7% and 70.4% for group C2. Among the most important metabolites discriminating initial remission (B) and sustained remission (C1) were acetate, choline and succinate (higher in B), and 2-hydroxyisobutyrate and pyruvate (higher in C1). Moreover, to a lesser extent, 2-hydroxybutyrate, 3-hydroxybutyrate, alanine, formate, glucose, glutamate and lactate were higher in B and glutamine and hypoxanthine were higher in C1 (**Figure 5C**). These results overlapped only in part with the weights obtained by comparing B and C2 (**Figure 5D**). In fact, the most relevant metabolites discriminating the B and C2 were glucose and succinate (accumulating in B), and pyruvate, 2-hydroxyisobutyrate, carnitine and acetylcarnitine (higher in C2). Similarly to the comparison between B and C1, 2-hydroxybutyrate, 3-hydroxybutyrate, and formate were somewhat higher in B compared to C2 while lysine was depleted. Moreover, alanine and hypoxanthine showed opposite trends to that observed above. The model built for the comparison of C1 and C2 led to results qualitatively similar in terms of discriminating metabolites. However, permutation tests lead to non-significant results and this can probably be partly attributed to the limited number of samples available for this comparison.

### Multivariate Statistical Analysis of NMR Spectra of Urine Samples

In marked contrast to the blood serum sample analyses, OSC-PLS-DA analysis of the urine samples determined that only the model built for the comparison of groups A and B (built using 2 classes and 2 LVs) had statistically significant predictivity (p∼0.039). The scores plots, weights plots and discriminatory metabolites for this model are provided in **Figure S3**.

### Carnitine and Acetylcarnitine in Active MM

**Figure 6:**
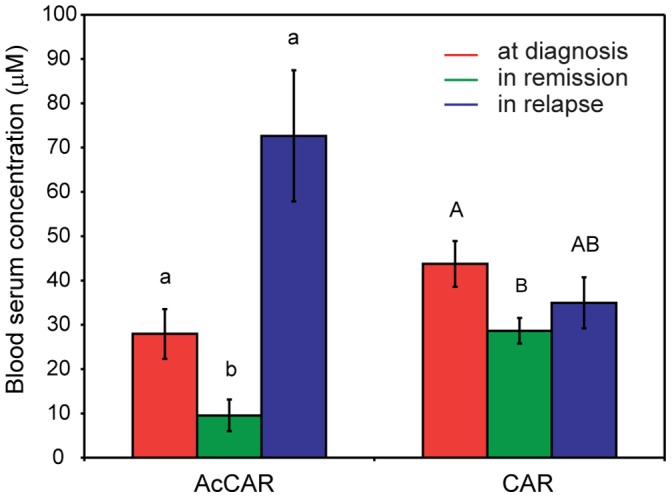
Blood serum concentration of acetylcarnitine and carnitine. Blood serum concentration of acetylcarnitine (AcCAR) and carnitine (CAR) in MM patients at diagnosis, in remission and after relapse of active disease following chemotherapy. Data shown as mean ± s.e.m. Statistical significance calculated according to Kruskal-Wallis one-way ANOVA (p<0.05).

OSC-PLS-DA of blood serum comparing patients in post treatment remission versus relapse (groups B and C2) identified carnitine and acetylcarnitine as discriminatory metabolites that increase on relapse. Interestingly, these metabolites were not powerful discriminators of the other serum sample classes. To further investigate the role of these candidate metabolites in differentiating patients with active disease from those in remission we quantified these two metabolites in all the serum samples (**Figure 6**). Receiver operating characteristic (ROC) curve for acetylcarnitine is reported in **Figure S4**. The statistical analysis revealed that carnitine and/or acetylcarnitine were indeed increased in samples from patients with active disease both before (group A) and after treatment (i.e. relapsed, group C2). Together these findings identify carnitine and acetylcarnitine as novel candidate blood serum biomarkers associated with active MM disease.

## Discussion

Recent studies have highlighted the potential of NMR-based metabolomics as a diagnostic and prognostic tool in disease, based on the analysis of human biofluids [15]–[23]. We have investigated the potential of a ^1^H NMR-based metabolomics analysis of human biofluids (blood serum and urine) to identify novel metabolic biomarkers indicative of the presence of active disease in MM patients. Owing to the availability of matched blood serum and urine samples collected in the context of the Myeloma IX trial, we were able to compare the effects induced on the metabolic profiles in blood serum and urine of MM patients carrying active disease at first diagnosis (prior to receiving chemotherapy), in disease remission after chemotherapy, and after the relapse to the active disease. Besides a ^31^P MRS study on phospholipids from sera of patients with MM [35], to the best of our knowledge, this paper represents the first study of global blood serum and urine-derived metabolites in the context of MM.

An untargeted metabolomics approach was used to assess changes across multiple metabolic pathways in active disease compared to disease remission after chemotherapy or after relapse to the active disease state. The results of our study indicate that the blood serum samples were the most beneficial to providing information on general metabolic changes that could be associated with active MM. Multivariate statistics was used to build models with significant predictive power among all the time-points of sample collection. On the contrary, for the urine samples, only modelling the comparison between patients at diagnosis and after treatment resulted in significant results. We can only speculate on the underlying cause and putatively attribute this result to the variable degree of renal failure affecting MM patients. This variability could in turn contribute to masking more subtle metabolic differences induced by the disease in patients with active disease compared to subjects that have achieved remission following chemotherapy.

The multivariate statistical analysis performed on the NMR spectra acquired on the blood serum samples of all the patients in this study seem to indicate that the overall metabolic changes induced by the disease on the blood serum profiles of these patients at first diagnosis and at the later time of disease relapse overlap only partially, as indicated in the OSC-PLS-DA scores plot in **Figure 1**. However, this is probably not so surprising due to the poor health status of these generally elderly and very sick patients at the time of their first diagnosis. For instance some of the most relevant metabolic changes in blood serum of patients at diagnosis and after the end of chemotherapy included the accumulation of blood serum creatinine and glucose in patients with active disease which are likely associated with the mild renal dysfunction suffered by MM patients. Also likely related to renal dysfunction is the mild accumulation of myo-inositol in the blood serum of patients at diagnosis compared to treated patients [36]. The complications induced by multiple myeloma on renal function and bone loss are likely to be less pronounced in patients with active disease after relapse due to faster medical intervention. The comparisons of the blood serum profiles of patients in remission and after disease relapse highlighted few candidate metabolites that could discriminate between patients still in remission and subjects with emerging relapsed disease. For instance 2-hydroxybutyrate gradually decreased in blood serum of patients after chemotherapy and reached the lowest levels in relapsed patients. Recently 2-hydroxybutyrate has been observed to accumulate in blood plasma of individuals at the early stages of diabetes [37] and in a non-diabetic population affected by insulin resistance and impaired glucose regulation [38].

Of particular interest was the observed accumulation of carnitine and/or acetylcarnitine in blood serum of MM patients both at diagnosis and after relapse, suggesting these metabolites as candidate blood serum biomarkers associated with active MM disease. Although there is no existing reported link between carnitine or acetylcarnitine with the pathobiology of MM, there have been reports that indicate that carnitine may promote antibody mediated immune responses either by enhancing plasma cell differentiation and/or by enhancing immunoglobulin (Ig) synthesis and secretion by plasma cells [39], [40]. Carnitine can be both absorbed from food intake and synthesized in the liver, kidney and brain [41], [42]. This metabolite has a key role in fatty acid metabolism and it is responsible for catalyzing the transport of acyl groups through the inner mitochondrial membrane for β-oxidation of long chain fatty acids. Carnitine palmitoyltransferase I (CPT-I) converts acyl-CoA and carnitine to acylcarnitine which is then transported to the inner mitochondrial matrix. CPT-II releases carnitine and the acyl group and the latter is further conjugated with CoA for β-oxidation [41], [43]. The increased levels of blood serum carnitine and, to a larger extent, acetylcarnitine in MM patients could therefore entail an increased lipid oxidation in highly metabolically active myeloma cells. The increased demand of lipid oxidation in tumour cells has been previously reported as a reason to avoid carnitine supplementation during anticancer therapy [44], [45]. The notion that the malignant plasma cells in MM are the direct source of elevated carnitine (and by extension its acetylcarnitine) is further supported by a second NMR based metabolomics study that identified increased carnitine release by mouse myeloma cells associated with culture in conditions that promoted enhanced antibody secretion [40].

It is interesting therefore to consider whether our studies have identified a potential new avenue of clinical intervention in MM. In this regard it is noteworthy that several agents have been proposed for use in combination therapy for the treatment of a wide range of tumours that either directly (e.g. through administration of etomoxir) or indirectly (e.g. via the administration of fatty acid synthase inhibitors) inhibit CPT-I [46], [47]. Indeed, the inhibition of CPT-I-regulated fatty acid β-oxidation promotes an up-regulation of ceramide levels which have been associated with a ceramide-mediated apoptotic pathway via the peroxisome proliferator-activated receptor (PPAR) gamma, induction of proapoptotic genes BNIP3, tumour necrosis factor (TNF)-related apoptosis-inducing ligand (TNSF10) and death-associated protein kinase 2 (DAPK2) [48], [49]. However, the biology of carnitine in MM may be still more complex because of several reports that the metabolite also positively regulates osteoblast activity and therefore in some patients may have beneficial retardation effects in MM associated osteolytic disease [50]–[52]. Our study therefore justifies an extended analysis of existing MM blood serum archives to further dissect and define the association of carnitine with MM and its clinical course and in particular its relative association with kidney and bone disease. An important aim of such a study would be to determine whether a signature can be derived for patients most likely to benefit from interventionist clinical trials.

Finally we reiterate that these discoveries of informative molecular differences between patient groups were derived from blood serum samples that were neither collected nor stored optimally. This provides evidence that existing patient material archives can be accessible to metabolomics analyses and that samples of this type should not simply be overlooked based upon community misconceptions on sample quality.

## Supporting Information

Figure S1 **Principal Component Analysis of NMR spectra acquired on blood serum and urine samples.** Scores plots obtained from PCA performed on the NMR spectra of 71 (19, 27, 10 and 15 samples for groups A, B, C1 and C2) blood serum (A) and 67 (21, 24, 10 and 12 samples for groups A, B, C1 and C2) urine samples (B). Group A (solid red, 21 samples): patients at diagnosis; group B (solid green, 24 samples): patients after chemotherapy; group C1 (solid blue, 10 samples): sustained remission; group C2 (empty blue, 15 samples): in relapse after chemotherapy.
(TIF)

Figure S2 **Partial Least Squares Discriminant Analysis** **of NMR spectra acquired on blood serum samples.** Scores (A and B) and weights (on LV1; C and D) plots obtained from OSC-PLS-DA performed on the NMR spectra of 29 and 34 blood serum samples for the comparison of A versus C1 (A and C) and A versus C2 (B and D). Group A (solid red, 19 samples): patients at diagnosis; group C1 (solid blue, 10 samples): sustained remission; group C2 (empty blue, 15 samples): in relapse after chemotherapy. H-xan: hypoxantine; Lac: lactate; Cre: creatinine; Suc: succinate; Pyr: pyruvate; Glu: glutamate; Gln: glutamine; Ace: acetate; 2HiB: 2-hydroxyisobutyrate.
(TIF)

Figure S3 **Partial Least Squares Discriminant Analysis** **of NMR spectra acquired on urine samples.** Scores (A) and weights (on LV1; B) plots obtained from OSC-PLS-DA performed on the NMR spectra of 45 urine samples. Group A (solid red, 21 samples): patients at diagnosis; group B (solid green, 24 samples): patients after chemotherapy. Form: formate; Hip: hippurate; Phe: phenylalanine; Bet: betaine; Gly: glycine; Tau: taurine; TMAO: trimethylamine N-oxide; Ace: acetate; Ala: alanine.
(TIF)

Figure S4 **Receiver operating characteristic curve for acetylcarnitine.** Area under the ROC curve is 0.81 (95% confidence interval, 0.70–0.91). Cutoff level derived from the ROC curve was 9.3 µM (sensitivity 82%, specificity 78%).
(TIF)
