# Supplementary figures and images for: Proton NMR-Based Metabolite Analyses of Archived Serial Paired Serum and Urine Samples from Myeloma Patients at Different Stages of Disease Activity Identifies Acetylcarnitine as a Novel Marker of Active Disease
Source: PLoS One. 2013 Feb 19;8(2):e56422. doi: 10.1371/journal.pone.0056422 (PMC3576408; doi:10.1371/journal.pone.0056422)

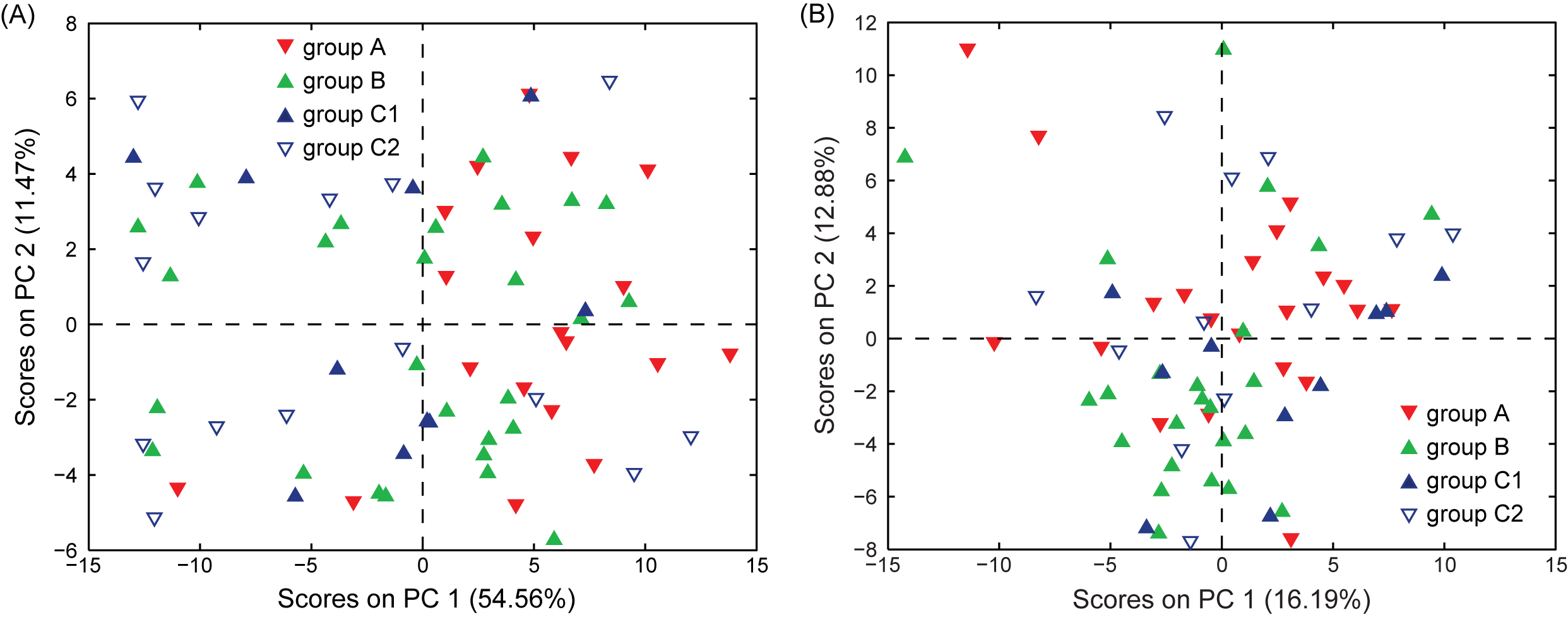

Supplement: Figure S1 — Principal Component Analysis of NMR spectra acquired on blood serum and urine samples. Scores plots obtained from PCA performed on the NMR spectra of 71 (19, 27, 10 and 15 samples for groups A, B, C1 and C2) blood serum (A) and 67 (21, 24, 10 and 12 samples for groups A, B, C1 and C2) urine samples (B). Group A (solid red, 21 samples): patients at diagnosis; group B (solid green, 24 samples): patients after chemotherapy; group C1 (solid blue, 10 samples): sustained remission; group C2 (empty blue, 15 samples): in relapse after chemotherapy. (TIF) [file pone.0056422.s001.tif]

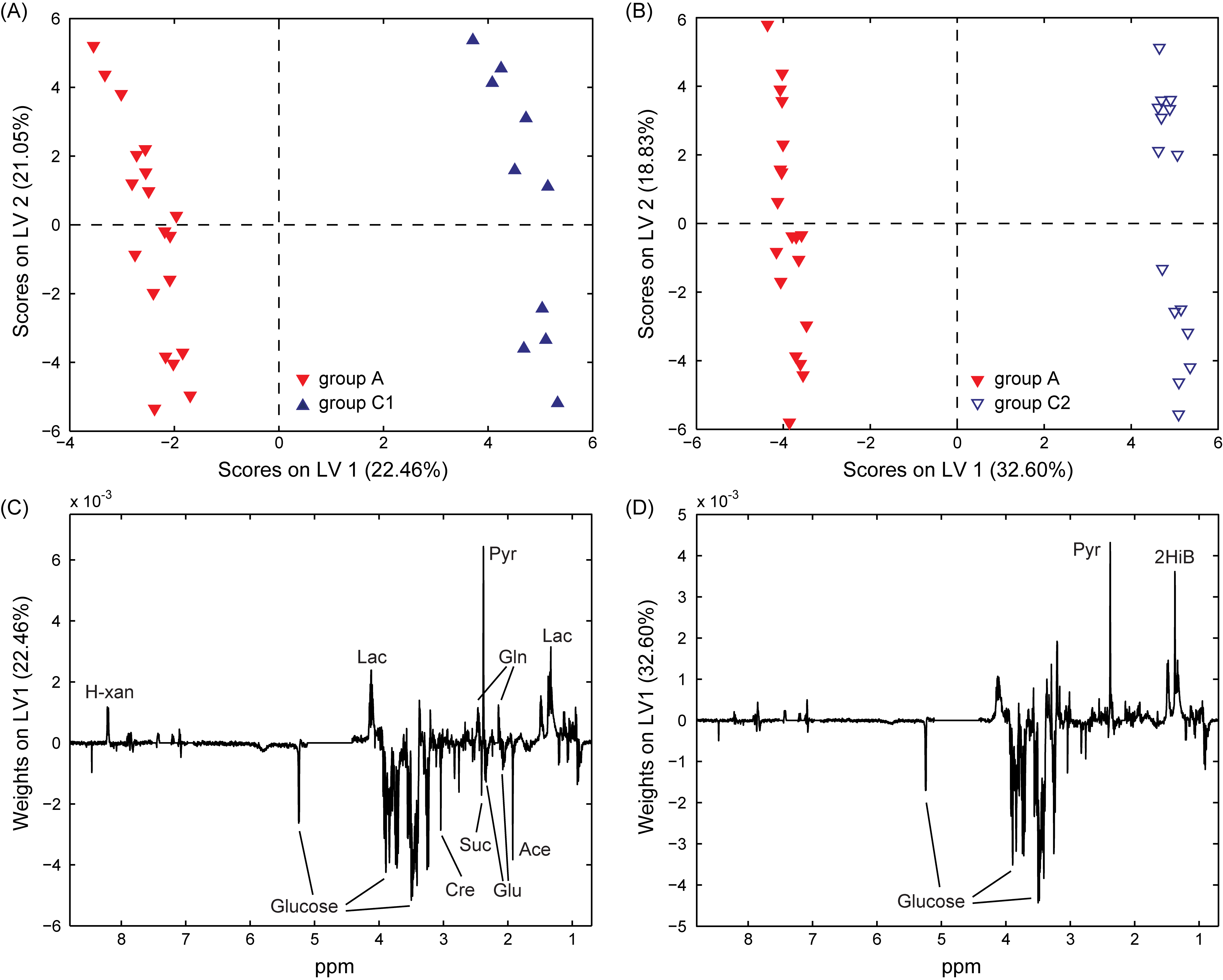

Supplement: Figure S2 — Partial Least Squares Discriminant Analysis of NMR spectra acquired on blood serum samples. Scores (A and B) and weights (on LV1; C and D) plots obtained from OSC-PLS-DA performed on the NMR spectra of 29 and 34 blood serum samples for the comparison of A versus C1 (A and C) and A versus C2 (B and D). Group A (solid red, 19 samples): patients at diagnosis; group C1 (solid blue, 10 samples): sustained remission; group C2 (empty blue, 15 samples): in relapse after chemotherapy. H-xan: hypoxantine; Lac: lactate; Cre: creatinine; Suc: succinate; Pyr: pyruvate; Glu: glutamate; Gln: glutamine; Ace: acetate; 2HiB: 2-hydroxyisobutyrate. (TIF) [file pone.0056422.s002.tif]

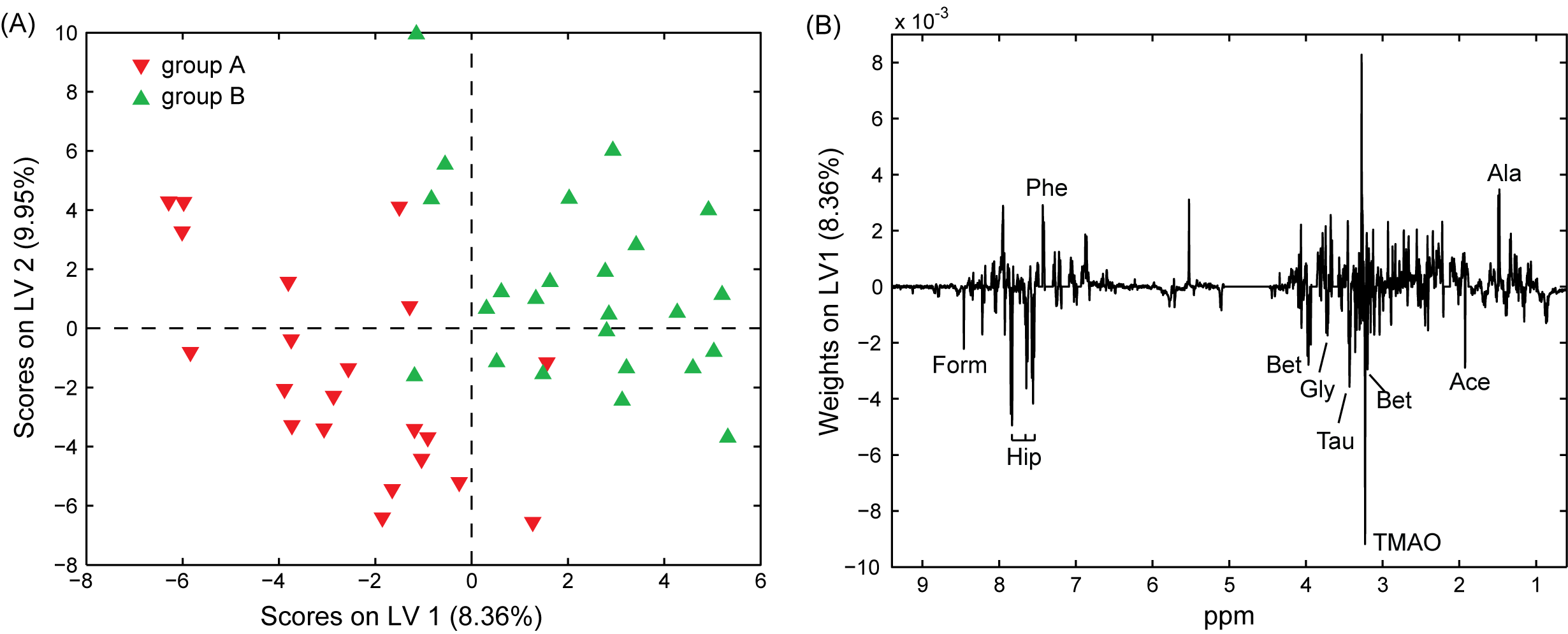

Supplement: Figure S3 — Partial Least Squares Discriminant Analysis of NMR spectra acquired on urine samples. Scores (A) and weights (on LV1; B) plots obtained from OSC-PLS-DA performed on the NMR spectra of 45 urine samples. Group A (solid red, 21 samples): patients at diagnosis; group B (solid green, 24 samples): patients after chemotherapy. Form: formate; Hip: hippurate; Phe: phenylalanine; Bet: betaine; Gly: glycine; Tau: taurine; TMAO: trimethylamine N-oxide; Ace: acetate; Ala: alanine. (TIF) [file pone.0056422.s003.tif]

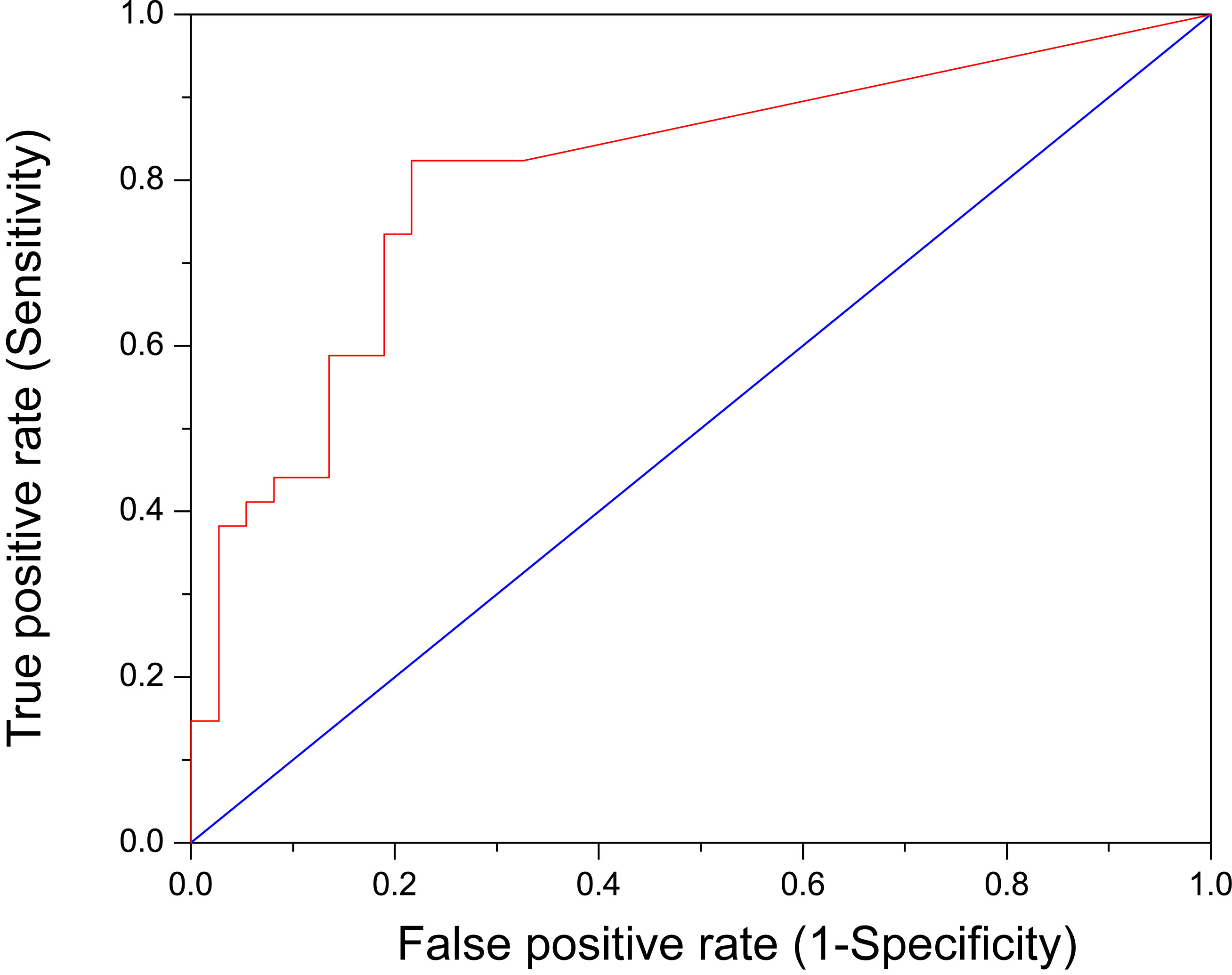

Supplement: Figure S4 — Receiver operating characteristic curve for acetylcarnitine. Area under the ROC curve is 0.81 (95% confidence interval, 0.70–0.91). Cutoff level derived from the ROC curve was 9.3 µM (sensitivity 82%, specificity 78%). (TIF) [file pone.0056422.s004.tif]
